# Supplementary material for: A Systematic Review of Intracellular Microorganisms within Acanthamoeba to Understand Potential Impact for Infection
Source: Pathogens. 2021 Feb 18;10(2):225. doi: 10.3390/pathogens10020225 (PMC7922382; doi:10.3390/pathogens10020225)
Supplement: Supplementary file 1 [file pathogens-10-00225-s001.zip › pathogens-1112508-proofed author-supple/S1_Newcastle-Ottawa Scale (NOS)_Systematic Review-BR, DS, HKP, MW, FLH & NC_UNSW, Sydney.docx]

**Table S1:** The Newcastle-Ottawa Scale (NOS) for assessing the quality of cross-sectional studies included in this review

| **S.N.** | **Study ID** | **Methodological quality (max 4 points)** | **Comparability of results (max 2 points)** | **Findings measures (max 3 points)** | **Raw score** |
| --- | --- | --- | --- | --- | --- |
|  | Fritsche et al. (1993), USA | **☆☆☆** | **☆** | **☆☆** | 6 |
|  | Yogita et al. (1995), Philippines | **☆☆☆** | **☆** | **☆☆** | 6 |
|  | Chung et al. (1997), South Korea | **☆☆☆** | **☆** | **☆☆** | 6 |
|  | Amann et al. (1997), Germany | **☆☆☆** | **☆☆** | **☆☆☆** | 8 |
|  | Michel et al. (1997), Germany | **☆☆☆** | **☆** | **☆☆☆** | 7 |
|  | Michel et al. (1998), Germany | **☆☆☆** | **☆** | **☆☆** | 6 |
|  | Hoffmann et al. (1998), Germany | **☆☆☆** | **☆** | **☆☆** | 6 |
|  | Molled et al. (1999), Germany | **☆☆☆** | **☆** | **☆☆** | 6 |
|  | Horn et al. (1999), Germany | **☆☆☆☆** | **☆☆** | **☆☆☆** | 9 |
|  | Fritsche et al. (1999), USA | **☆☆☆☆** | **☆☆** | **☆☆☆** | 8 |
|  | Fritsche et al. (2000), USA | **☆☆☆☆** | **☆☆** | **☆☆☆** | 9 |
|  | Birtles et al. (2000), Greece | **☆☆☆☆** | **☆☆** | **☆☆☆** | 9 |
|  | Horn et al. (2001), Germany | **☆☆☆☆** | **☆☆** | **☆☆☆** | 9 |
|  | Horn et al. (2002), Germany | **☆☆☆☆** | **☆☆** | **☆☆** | 8 |
|  | Scola et al. (2003), France | **☆☆☆** | **☆** | **☆☆** | 6 |
|  | Yu et al. (2007), South Korea | **☆☆☆☆** | **☆☆** | **☆☆☆** | 9 |
|  | Xuan et al. (2007), South Korea | **☆☆☆☆** | **☆☆** | **☆☆☆** | 9 |
|  | Heinz et al. (2007), Austria | **☆☆☆☆** | **☆☆** | **☆☆☆** | 9 |
|  | Lorenzo-Morales et al. (2007), Spain | **☆☆** | **☆** | **☆☆☆** | 6 |
|  | Scheid et al. (2008), Germany | **☆☆** | **☆☆** | **☆☆** | 6 |
|  | Schmitz-Esser et al. (2008), Austria | **☆☆☆** | **☆☆** | **☆☆☆** | 8 |
|  | Choi et al. (2009), South Korea | **☆☆** | **☆☆** | **☆☆☆** | 7 |
|  | Matsuo et al. (2010), Japan | **☆☆☆☆** | **☆☆** | **☆☆☆** | 9 |
|  | Iovieno et al. (2010), USA | **☆☆☆☆** | **☆☆** | **☆☆☆** | 9 |
|  | Corsaro et al. (2010), Spain | **☆☆☆** | **☆☆** | **☆☆☆** | 8 |
|  | Cohen et al. (2011), France | **☆☆☆☆** | **☆☆** | **☆☆☆** | 9 |
|  | Glaser et al. (2011), USA | **☆☆☆** | **☆☆** | **☆☆☆** | 8 |
|  | Gaze et al. (2011), UK | **☆☆☆** | **☆** | **☆☆** | 6 |
|  | Corsaro et al. (2013), Germany | **☆☆☆☆** | **☆☆** | **☆☆☆** | 9 |
|  | Sampo et al. (2014), Japan | **☆☆☆** | **☆** | **☆☆☆** | 7 |
|  | Lagkouvardos et al. (2014), Austria | **☆☆☆☆** | **☆☆** | **☆☆☆** | 9 |
|  | Maschio et al. (2015), Brazil | **☆☆☆** | **☆☆** | **☆☆☆** | 8 |
|  | Maschio et al. (2015), Brazil | **☆☆** | **☆☆** | **☆☆** | 6 |
|  | Nakagawa et al. (2015), Japan | **☆☆☆** | **☆☆** | **☆☆** | 7 |
|  | Niyyati et al. (2015), Iran | **☆☆☆☆** | **☆** | **☆☆☆** | 8 |
|  | Magnet et al. (2015), Spain | **☆☆☆☆** | **☆** | **☆☆☆** | 8 |
|  | Purssell et al. (2016), Canada | **☆☆☆☆** | **☆** | **☆☆☆** | 8 |
|  | Fukumoto et al. (2016), Japan | **☆☆** | **☆** | **☆☆☆** | 6 |
|  | Muller et al. (2016), Austria | **☆☆☆** | **☆☆** | **☆☆☆** | 8 |
|  | Scheikl et al. (2016), Austria | **☆☆☆☆** | **☆** | **☆☆☆** | 8 |
|  | Faizah et al. (2017), Malaysia | **☆☆☆☆** | **☆☆** | **☆☆☆** | 9 |
|  | Chan et al. (2018), Malaysia | **☆☆☆** | **☆☆** | **☆☆☆** | 8 |
|  | Hajialilo et al. (2019), Iran | **☆☆☆☆** | **☆☆** | **☆☆☆** | 9 |
