# Supplementary material for: A Systematic Review of Intracellular Microorganisms within Acanthamoeba to Understand Potential Impact for Infection
Source: Pathogens. 2021 Feb 18;10(2):225. doi: 10.3390/pathogens10020225 (PMC7922382; doi:10.3390/pathogens10020225)
Supplement: Supplementary file 1 [file pathogens-10-00225-s001.zip › pathogens-1112508-proofed author-supple/S2_List of Figures_Systematic Review-BR, DS, HKP, MW, FLH & NC_UNSW, Sydney.docx]

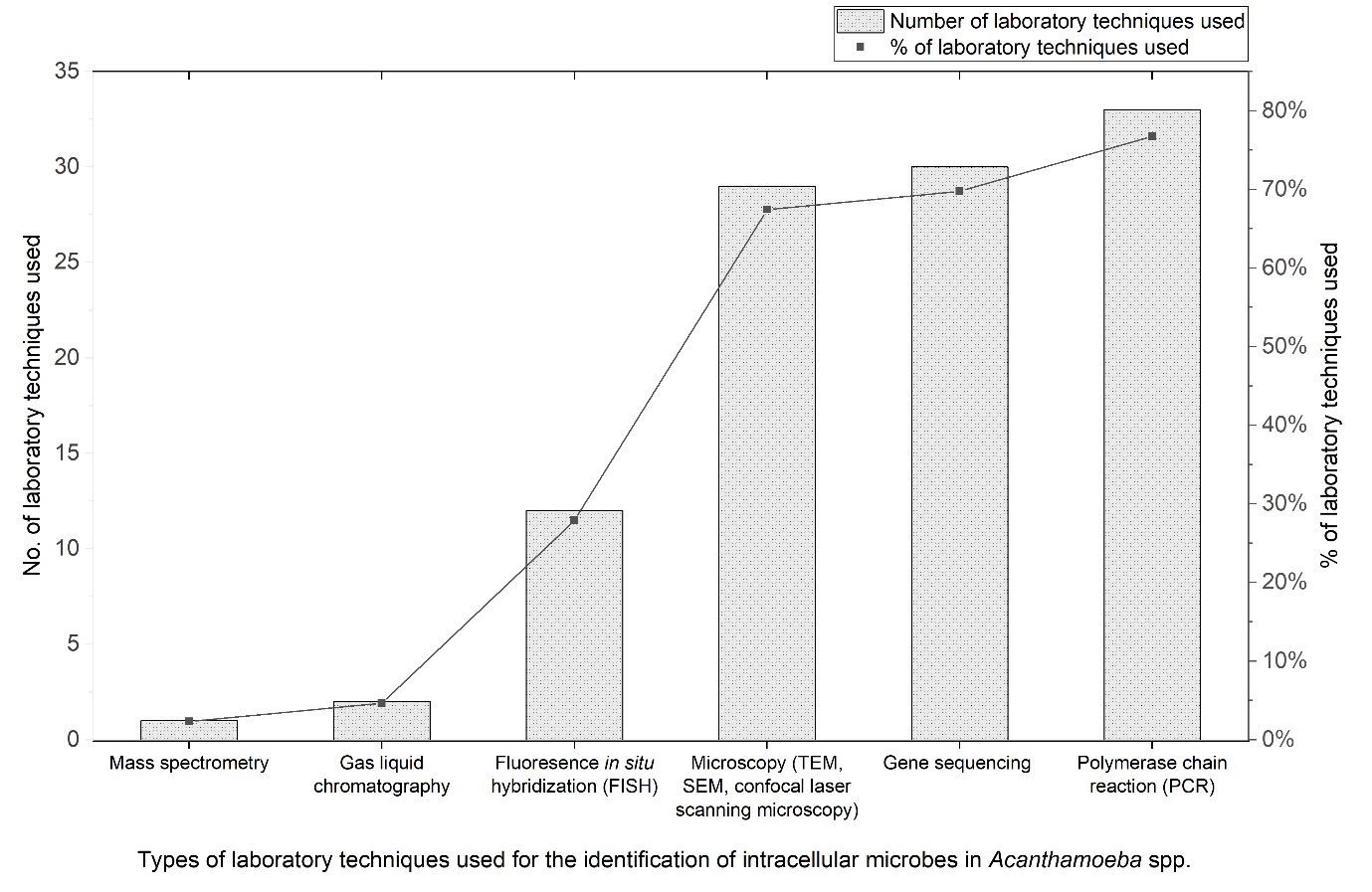


**Figure S1:** Laboratory techniques used for identification of intracellular microbes in *Acanthamoeba* spp.


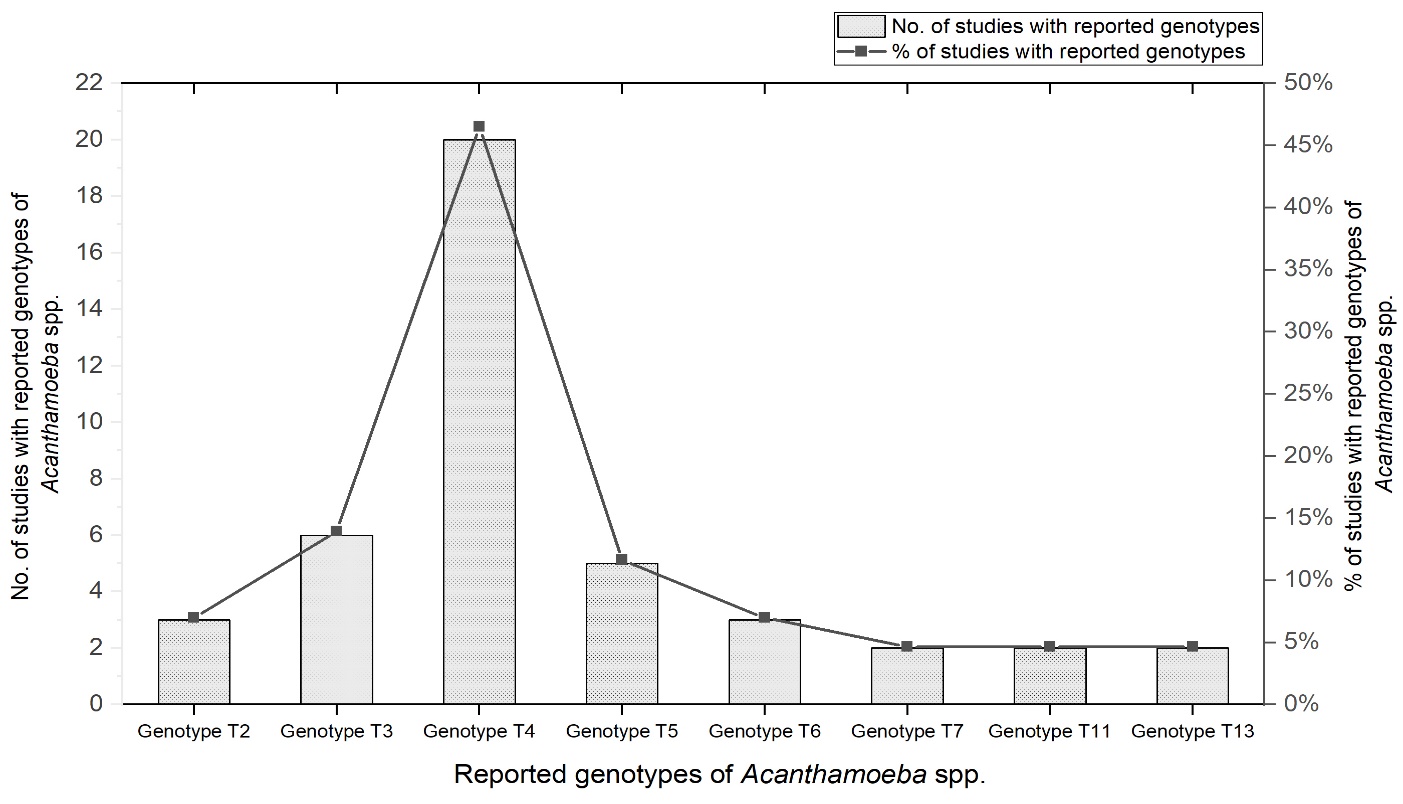


**Figure S2:** Reported genotypes of *Acanthamoeba* spp.


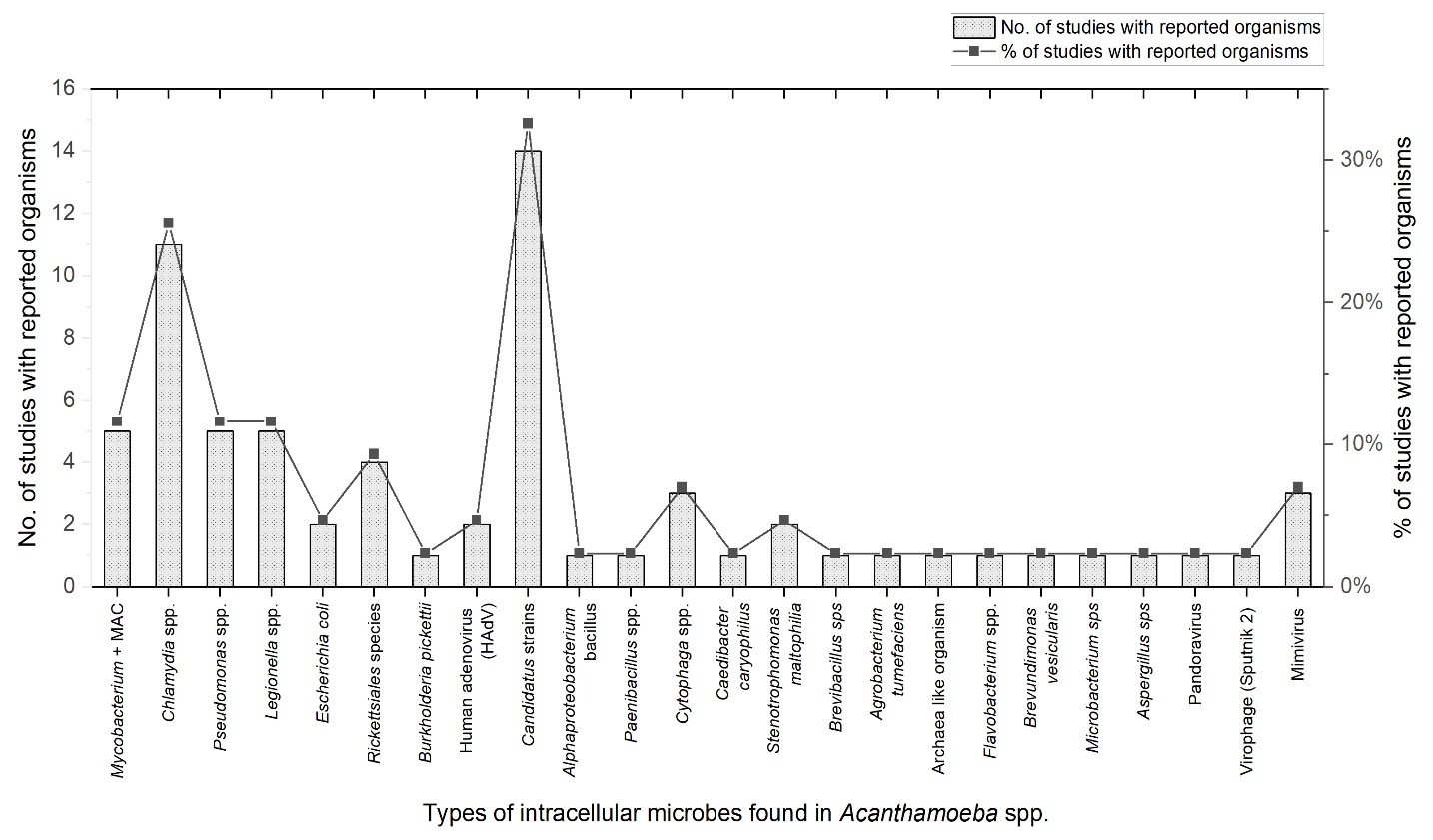


**Figure S3:** Types of intracellular prokaryotes identified in *Acanthamoeba* spp.


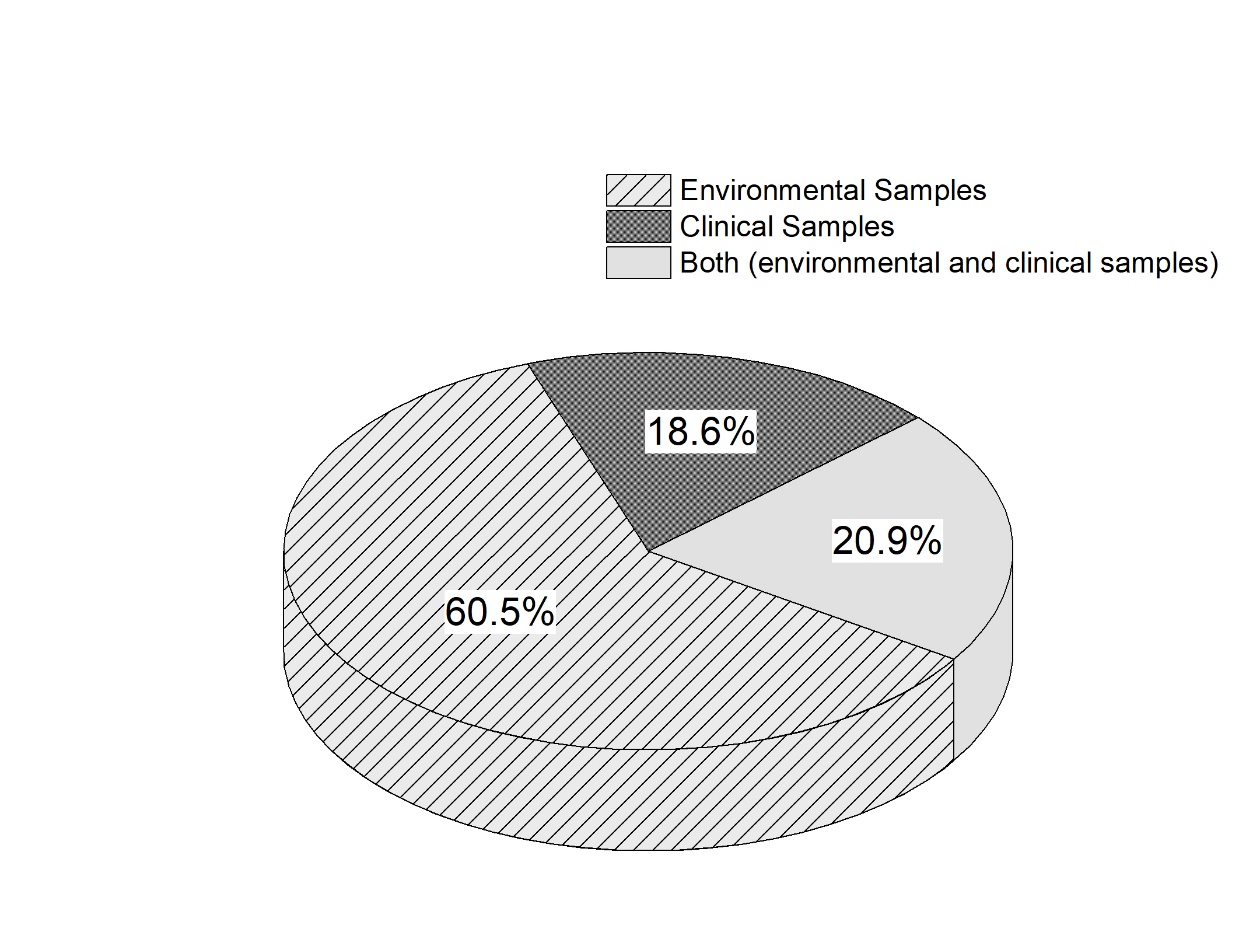


**Figure S4:** Types of analysed samples
